# Natural History and Ecology of Interactions Between *Bordetella* Species and Amoeba

**DOI:** 10.3389/fcimb.2022.798317

**Published:** 2022-02-09

**Authors:** Longhuan Ma, Bodo Linz, Amanda D. Caulfield, Kalyan K. Dewan, Israel Rivera, Eric T. Harvill

**Affiliations:** ^1^ Department of Infectious Diseases, College of Veterinary Medicine, University of Georgia, Athens, GA, United States; ^2^ Division of Microbiology, Department of Biology, Friedrich Alexander University Erlangen-Nuremberg, Erlangen, Germany

**Keywords:** *Bordetella* species, *Dictyostelium discoideum*, interactions, evolution, GWAS -genome-wide association study

## Abstract

A variety of bacteria have evolved the ability to interact with environmental phagocytic predators such as amoebae, which may have facilitated their subsequent interactions with phagocytes in animal hosts. Our recent study found that the animal pathogen *Bordetella bronchiseptica* can evade predation by the common soil amoeba *Dictyostelium discoideum*, survive within, and hijack its complex life cycle as a propagation and dissemination vector. However, it is uncertain whether the mechanisms allowing interactions with predatory amoebae are conserved among *Bordetella* species, because divergence, evolution, and adaptation to different hosts and ecological niches was accompanied by acquisition and loss of many genes. Here we tested 9 diverse *Bordetella* species in three assays representing distinct aspects of their interactions with *D. discoideum*. Several human and animal pathogens retained the abilities to survive within single-celled amoeba, to inhibit amoebic plaque expansion, and to translocate with amoebae to the fruiting body and disseminate along with the fruiting body. In contrast, these abilities were partly degraded for the bird pathogen *B. avium*, and for the human-restricted species *B. pertussis* and *B. parapertussis*. Interestingly, a different lineage of *B. parapertussis* only known to infect sheep retained the ability to interact with *D. discoideum*, demonstrating that these abilities were lost in multiple lineages independently, correlating with niche specialization and recent rapid genome decay apparently mediated by insertion sequences. *B. petrii* has been isolated sporadically from diverse human and environmental sources, has acquired insertion sequences, undergone genome decay and has also lost the ability to interact with amoebae, suggesting some specialization to some unknown niche. A genome-wide association study (GWAS) identified a set of genes that are potentially associated with the ability to interact with *D. discoideum*. These results suggest that massive gene loss associated with specialization of some *Bordetella* species to a closed life cycle in a particular host was repeatedly and independently accompanied by loss of the ability to interact with amoebae in an environmental niche.

## Introduction

The genus *Bordetella* comprises 16 named species, including the “classical” species consisting of *B. bronchiseptica*, *B. pertussis* and *B. parapertussis*. The classical *Bordetella* species are found in mammalian hosts, as *B. pertussis* infects humans, the two lineages of *B. parapertussis* infect either humans (*B. parapertussis*_hu_) or sheep (*B. parapertussis*_ov_), and *B. bronchiseptica* is a pathogen of a wide range of mammals (Mattoo and Cherry, 2005). Other, more distantly related species, often referred to as the non-classical *Bordetella* species, cause infections in a variety of hosts, including *B. holmesii* in humans (Weyant et al., 1995), *B. avium* and *B. hinzii* in birds (Raffel et al., 2002; Register and Kunkle, 2009), and *B. pseudohinzii* in rodents (Ivanov et al., 2015; Ivanov et al., 2016). *B. trematum* and *B. ansorpii* have been isolated from infected wounds of immunocompromised patients (Vandamme et al., 1996; Ko et al., 2005), and *B. bronchialis*, *B. flabilis* and *B. sputigena* from respiratory samples of patients with cystic fibrosis (Vandamme et al., 2015). In addition to animal hosts, some *Bordetella* species have been isolated from environmental sources. *B. petrii* has been isolated from multiple natural environments, including an anaerobic, dechlorinating bioreactor culture enriched by river sediment, marine sponges and grass root consortia (von Wintzingerode et al., 2001).

Due to our anthropocentric view and a strong research bias toward pathogens relevant to human or mammalian disease, the animal-derived *Bordetella* species are well-characterized. However, more diverse *Bordetella* species have been isolated from soil and water, and their genetic analysis suggested that current mammalian *Bordetella* species evolved from ancestral lineages of environmental origin. (Hamidou Soumana et al., 2017). Phylogenetic comparison of 16S rRNA sequences of bacteria isolated from various environmental sources, including soil, sediment, water, and plant roots, showed that environmental *Bordetella* species possess a significantly higher genetic diversity than those from human- and animal-associated samples. Sequences from animal-associated samples were confined to only four sequence clusters near the top of the phylogenetic tree, in contrast to environmental samples that were present in all ten identified sequence clusters, including near the tree root. The branching of the phylogeny suggested an environmental origin of the genus *Bordetella* and successive adaptation of some lineages and species to animal hosts (Hamidou Soumana et al., 2017).

A whole genome comparison of 128 genomes from 9 *Bordetella* species revealed that speciation and host adaptation was accompanied by substantial gene loss and gene acquisition (Linz et al., 2016). In particular, the evolution of human-restricted pathogens from host generalists, such as the origin of *B. parapertussis* and *B. pertussis* from *B. bronchiseptica*-like ancestors, was associated with large scale gene loss that resulted in genomes over 0.5 Mb (*B. parapertussis*) and over 1.2 Mb (*B. pertussis*) smaller than that of the apparent *B. bronchiseptica*-like ancestor. Genome reduction appears to have been mediated by homologous recombination between multiple identical copies of insertion sequence (IS) elements in the genome (Parkhill et al., 2003). The classical *Bordetella* form a distinct clade in a phylogenetic tree, and as a result of the relatively recent speciation events, their genomes are very closely related to each other. In contrast, the five species *B. holmesii*, *B. hinzii*, *B. pseudohinzii*, *B. avium*, and *B. trematum* that form the sister clade in the whole genome-based tree are phylogenetically more diverse (Ivanov et al., 2016; Linz et al., 2016).

*B. pertussis*, *B. parapertussis* and *B. bronchiseptica* have been found surviving inside various mammalian cell types *in vitro* and *in vivo*, including macrophages, neutrophils, dendritic cells, and epithelial cells (Steed et al., 1991; Friedman et al., 1992; Guzman et al., 1994; Lamberti et al., 2013; Bendor et al., 2015; Rivera et al., 2019). An *in vitro* assay showed that *B. bronchiseptica* appeared in the cytoplasm of infected epithelial cells while *B. pertussis* did not, indicating that different invasion and persistence strategies may be used by the two species (Schipper et al., 1994). Different virulence factors, such as adenylate cyclase toxin, filamentous hemagglutinin and pertussis toxin, have been reported to contribute to the ability to survive within mammalian cells (Masure, 1993; Ishibashi et al., 2001; Schaeffer and Weiss, 2001). Our recent study on intracellular survival of *Bordetella* spp. inside RAW 264.7 macrophages, a macrophage-like cell line derived from BALB/c mice, showed that bacterial survival and persistence is not restricted to the three classical *Bordetella* species. *B. hinzii*, *B. pseudohinzii*, and *B. trematum* survived inside macrophages at a ratio similar to that of *B. bronchiseptica*, suggesting that the mechanisms employed for intracellular survival are conserved among *Bordetella* species (Rivera et al., 2019). RNA-Seq revealed that phagocytosis by macrophages triggered a general stress response that activated genes involved in DNA repair, protein repair, oxidative stress response, and genes that encode enzymes for specific metabolic pathways in the internalized bacteria. Comparative genome analyses showed that the vast majority of those genes are indeed highly conserved among the individual *Bordetella* species (Rivera et al., 2019).

Various infectious pathogens have been found in free living amoeba (Molmeret et al., 2005; Guimaraes et al., 2016). These amoeba resistant microorganisms (ARMs) were shown to possess higher gene numbers and larger DNA contents compared to their relatives, suggesting that the interactions of pathogens with amoebae may pose a selective pressure on ARMs to acquire the genes that enable intracellular survival. In other words, as some authors put it, amoebae may function as “a training ground for bacterial intracellular survival” (Molmeret et al., 2005). Our previous study reported that *B. bronchiseptica* can survive inside the common soil amoeba *D. discoideum*, translocate to and propagate in the sori, and hijack the amoeba life cycle as a dissemination vector for bacterial transmission (Taylor-Mulneix et al., 2017a). Considering the similarities between amoebae and mammalian phagocytes, interactions between environmental bacteria and amoebae may represent the starting point for bacterial adaption to mammalian hosts (Taylor-Mulneix et al., 2017b). The *Bordetella* species have diverged, evolved, and adapted to different hosts and ecological niches, which was accompanied by loss and gain of multiple genes, including large scale gene loss during specialization to a single (human) host (Linz et al., 2016). However, whether the mechanisms allowing interaction with predatory amoebae are conserved among the *Bordetella* genus or are specific for some *Bordetella* species is unknown.

In this study, we tested nine *Bordetella* species in assays representing three separate aspects of their interactions with *D. discoideum*: intracellular survival in single-celled amoebae, inhibition of amoebic plaque expansion, and bacterial translocation to the fruiting bodies. Most animal-pathogenic *Bordetella* species were able to resist predation by amoebae and to propagate in sori. But these abilities were degraded for two human-restricted species, *B. pertussis* and *B. parapertussis*, the bird pathogen *B. avium*, and the natural environment isolated *B. petrii*. Comparative genome analyses allowed the identification of a set of genes that are associated with the ability of the bacteria to interact with *D. discoideum*. The majority of genes identified by GWAS were categorized as metabolic enzymes, transporters, expression regulators, or genes associated with general stress response. The diversity of gene types identified suggests that multiple bacterial mechanisms are involved in facilitating intracellular survival in amoebae. A positive correlation was observed between the ability of *Bordetella* spp. to survive inside amoebae and in mammalian phagocytes. This suggests that some genes involved in surviving amoebic predation may also facilitate *Bordetella* spp. survival within host macrophages, thereby evading host immunity.

## Materials and Methods

### Bacterial Strains and Growth

*B. bronchiseptica* strain RB50, *B. pertussis* strain 536, a streptomycin resistant variant of Tohama I, *B. parapertussis* ovine strain Bpp5 (*B. parapertussis*_ov_), *B. parapertussis* human strain 12822 (*B. parapertussis*_hu_), *B. pseudohinzii* strain 8-296-03, B. hinzii strain L60, *B. petrii* strain DSM12804, *B. avium* strain 197N, *B. holmesii* strain 04P3421 and *B. trematum* strain H044680328 were grown and maintained on BG agar (Difco) supplemented with 10% defibrinated sheep’s blood (Hema Resources). Liquid cultures were grown overnight at 37°C to mid-log phase (OD ∼0.6) in Stainer Scholte (SS) liquid broth. *Klebsiella pneumoniae* (*K. pneumoniae*) was grown at 37°C and maintained at 4°C on Luria-Bertani (LB) agar (Difco) and liquid cultures were grown at 37°C to mid-log phase in LB broth (Difco) (Carilla-Latorre et al., 2008; Taylor-Mulneix et al., 2017a). To test the proliferation rates of *Bordetella* species under 21°C, a temperature used in plaque formation assay, a subset of each *Bordetella* species (~10^7^ CFU) was inoculated in SS liquid broth and growth curves were measured with three replicates.

### Amoeba Strains and Growth

*D*. *discoideum* strain AX4 was used in this study. The unicellular amoebae were cultured in HL5 medium at 21°C and subcultured twice a week in fresh medium to prevent the cultures from reaching confluency. They were also grown on bacterial lawns as described below.

### Intracellular Survival Assay in *D. discoideum*

*D*. *discoideum* cells were grown to 80% confluency (~1 x 10^5^ CFU/well) in HL/5 medium in 96-well tissue culture plates (Greiner Bio-One) at 21°C. 10^5^ CFU of bacteria in 10 μl were added to each well, which corresponds to a multiplicity of infection (MOI) of 1. Plates were centrifuged at 300 x g for 10 minutes at room temperature and then incubated at 21°C. After 45 minutes, the supernatants of wells were replaced with 100 μl of 300 μg/ml gentamicin solution (Sigma-Aldrich) in HL/5 to kill extracellular bacteria, and plates were incubated at 21°C. At 2 hours post gentamicin treatment, the wells were washed three times with PBS to remove the antibiotic. To lyse the amoebae, 100 μl of 0.1% Triton-X solution was added, followed by a 5-minute incubation and subsequent vigorous pipetting and vortexing. To enumerate the intracellular bacteria, serial dilutions of the lysates were plated onto BG agar plates, and colony numbers were counted after incubation at 37°C for two to four days. As control, similar numbers of each species were cultured with media containing gentamicin without amoebae. After 2 hours, serial dilution of the solution were plated on BG agar for bacterial enumeration.

### Transcriptional Analysis of *B. bronchiseptica* Inside Vegetative Amoebae

*B. bronchiseptica* were incubated with unicellular *D. discoideum* as above, and a subset of the bacteria were incubated in HL/5 medium without amoeba as the negative control. After 2 h of gentamicin treatment, the HL/5 was removed, the unicellular amoebae were washed with PBS, and the samples were suspended in 1 ml of TRIzol for RNA extraction. RNA was extracted from the lysates using TRIzol (Ambion) and the Bacterial RNA isolation Kit (Max Bacterial Enhancement Reagent, Ambion) with implemented PureLink DNase treatment (Invitrogen) according to the manufacturer’s protocol. Quality assessed RNA samples were submitted in duplicates for Illumina sequencing at the Molecular Research Laboratory in Shallowater, TX, USA. Preparation of the Illumina sequencing library included depletion of ribosomal RNA from each sample. Filtering of low-quality reads and trimming of Illumina library adapters were performed using FASTQC and TRIMMOMATIC. High quality reads were mapped to the genome of *B. bronchiseptica* RB50 genome assembly NC_002927.3 using “Bowtie2”. Differential gene expression between two duplicates each of intracellular *B. bronchiseptica* and controls were evaluated using the “EdgeR” package for R implemented in the Bioconductor project. Raw data files, processed data files together with a metadata spreadsheet have been deposited in GEO. The accession number for the deposited data is GSE190363.

### Intracellular Survival Inside Macrophages

RAW 264.7 macrophages cells were grown to 80% confluency (∼1 × 10^5^ CFU/well) in Dulbecco’s Modified Eagle Medium (DMEM) supplemented with 10% FBS, glucose and glutamine in 48-well tissue-culture plates at 37°C. Bacteria were added in 10 μl PBS containing 10^5^ CFU (MOI of 1) as indicated. Plates were centrifuged at 300 x *g* for 10 minutes at room temperature and incubated at 37°C for 1 hour, after which gentamicin solution (Sigma-Aldrich) was added to a final concentration of 300 μg/ml to kill extracellular bacteria. Plates were incubated at 37°C for an additional 2 hours and subsequently washed with PBS. 0.1% Triton-X solution was administered, followed by a 5-minute incubation and vigorous pipetting and vortexing to lyse the macrophages. The samples were serially diluted and plated on BG agar plates to quantify total bacteria numbers.

### Predation Resistance Assay

Bacterial isolates grown on BG agar plates or LB agar plates were resuspended in 1 mL of PBS, plated on SM/5 agar plates, and incubated at 37°C to obtain bacterial lawns. *D. discoideum* cells were resuspended in fresh HL5 medium, counted in a hemacytometer chamber, and serially diluted in HL5 to cell concentrations of 5 cells per 1 μl. The bacterial lawns were spotted with 10 μl of the *D. discoideum* dilution. The plates were incubated at 21°C for 16 days. The area of plaques formed on bacterial lawns was measured at various time points.

### Enumeration of *Bordetella* spp. in *D. discoideum* Sori

To determine the number of *Bordetella* spp. in sori, individual sori grown on lawns of the appropriate species were collected using a pipet tip and transferred into a tube containing 100 μL PBS. The samples were vortexed vigorously to disrupt the fruiting body, and serial dilutions were plated onto BG agar plates. Colony numbers were counted after two to four days incubation at 37°C.

### Genome Comparisons and Protein Similarity Analysis

For comparison between “classical *bordetellae*”, the genes and predicted proteins of *B. bronchiseptica* strain RB50 (NC_002927.3), *B. parapertussis*_ov_ strain BPP5 (NC_018828.1), *B. parapertussis*_hu_ strain 12822 (NC_002928.3), and *B. pertussis* strain Tohama I (NC_002928.2) were compared using mGenomeSubtractor (Shao et al., 2010) and using the Artemis Comparison Tool (ACT) (Carver et al., 2008). Pseudogenes containing mutations that would prevent complete translation (premature stop codon, frame shift, truncation) were identified by direct pairwise comparisons between *B. bronchiseptica* RB50 and the other three genomes. Protein similarities of *B. bronchiseptica* genes and their homologs in the “non-classical *Bordetellae*” were determined as pairwise BLASTp comparisons as previously described (Rivera et al., 2019). Total protein sequences were extracted from NCBI for *B. bronchiseptica* strain RB50 (RefSeq assembly accession: GCF_000195675.1), *B. hinzii* strain L60 (GCF_000657715.1), *B. pseudohinzii* strain 8-296-03 (GCF_000657795.2), *B. avium* strain 197N (GCF_000070465.1), *B. petrii* strain DSM12804 (GCF_000067205.1), *B. trematum* strain H044680328 (GCF_900078695.1), and *B. holmesii* strain 04P3421 (GCF_000662215.1). Similarities between *B. bronchiseptica* proteins and their corresponding homologs in the “non-classical *bordetellae*” were calculated in mGenomeSubtractor as the H value for each protein, defined as the product of the highest BLASTp identity score ‘i’ and the length of the matching sequence length ‘lm’, divided by the query length ‘lq’ (H = i x lm/lq). Based on our previous work (Rivera et al., 2019), homologs of *B. bronchiseptica* genes with a protein similarity value of H ≥ 0.5 were considered to be present. Proteins with values of H ≥ 0.5 were validated as true orthologs by pairwise tBLASTx genome comparisons in ACT.

### Statistical Analysis

The mean ± standard error (error bars in figures) was determined for all appropriate data. The Pearson correlation coefficient was measured to determine the correlation between *Bordetella* spp. survival inside *D. discoideum* and RAW macrophages. GraphPad Prism version 6.04 was used to conduct these statistical tests and to generate figures.

## Results

### Human-Restricted Classical *Bordetella* Species Fail to Survive Within *D. discoideum*

**Figure 1:**
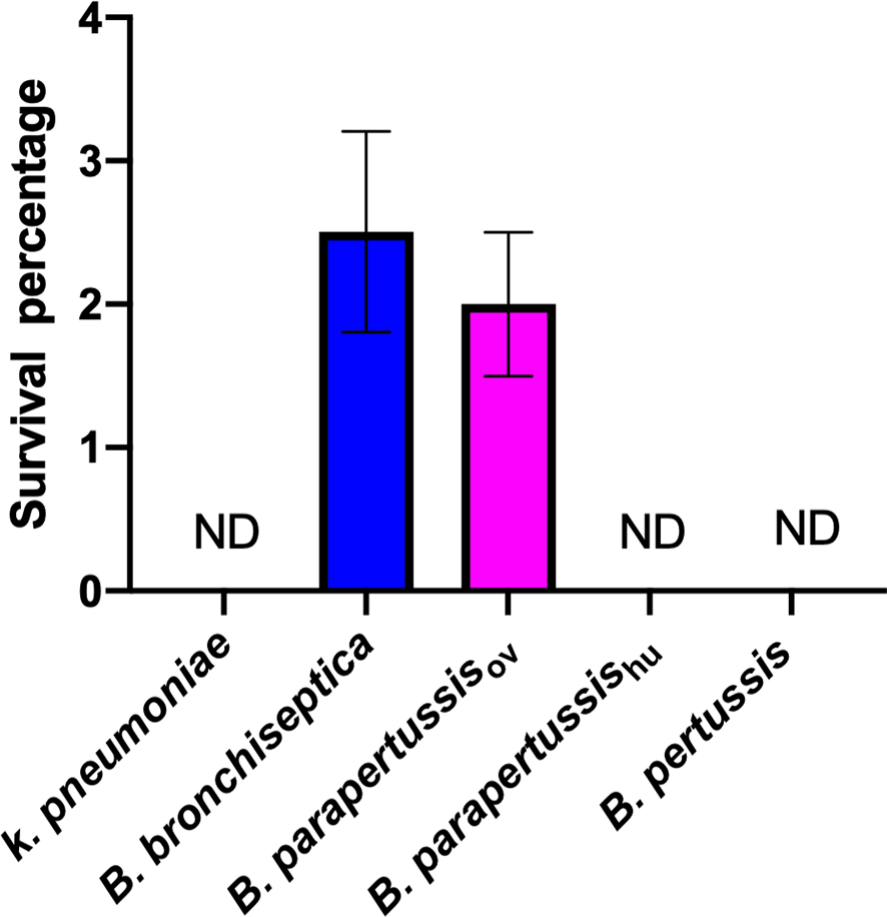
Human-restricted classical *Bordetella* species, *B. pertussis* and *B. parapertussis* human strain, fail to survive inside vegetative amoebae. Intracellular survival of three “classical” *Bordetella* species inside single cell amoeba was assessed in gentamicin protection assays. There were 3 replicates in each group. Error bars show the standard error of the mean. ND, none detected.

Our group has previously reported the ability of *B. bronchiseptica* to survive inside *D. discoideum* (Taylor-Mulneix et al., 2017a). However, whether this capacity is conserved among the other classical *Bordetella* species is unknown. To compare the intracellular survival of classical *Bordetella* species inside *D. discoideum*, a gentamicin protection assay was performed after exposure of *B. bronchiseptica*, *B. pertussis*, *B. parapertussis*_hu_, *B. parapertussis*_ov_ or *K. pneumoniae*, a food resource bacterium of amoeba, to a confluent monolayer of *D. discoideum* for 45 minutes. After 2 hours of gentamicin treatment, viable intracellular bacteria were enumerated by plating the lysed amoebae onto BG agar plates and counting the number of colonies after incubation at 37°C for two to four days. In contrast to *K. pneumoniae*, which was undetectable in this assay, more than two thousand viable *B. bronchiseptica* and *B. parapertussis*_ov_ cells were consistently recovered from the unicellular amoeba, which represented over two percent of the inoculated bacteria. However, *B. pertussis* and *B. parapertussis*_hu_ failed to survive inside *D. discoideum* (**Figure 1**), suggesting that during their adaptation to a closed life cycle in humans and the accompanying genome decay these species have lost some genes that are necessary to survive inside predatory amoebae.

### *D. discoideum* Efficiently Feeds on Human-Restricted *Bordetella* spp.

**Figure 2:**
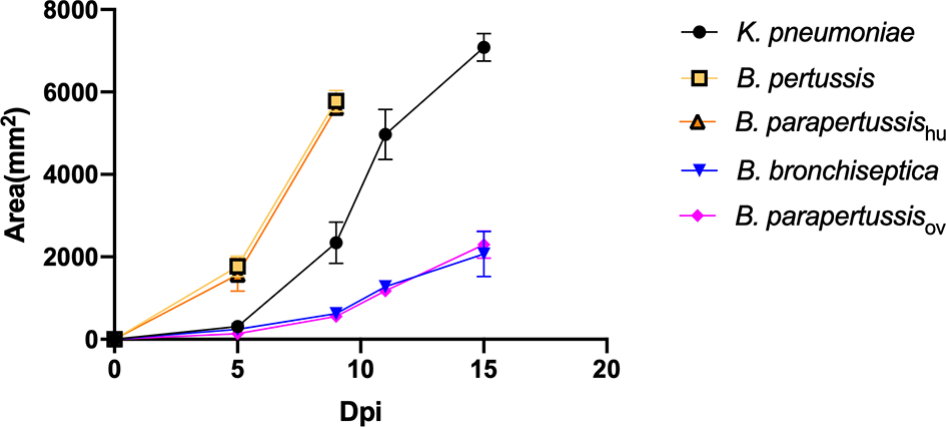
*D. discoideum* efficiently feed on human-restricted *Bordetella* species, *B. pertussis* and *B. parapertussis* human strain. The plaque areas formed on the lawn of *Bordetella* species were measured at various days post inoculation (Dpi). The plaques formed on the lawn of *B. pertussis* and *B. parapertussis_hu_* covered the entire plate before d15pi, therefore the last data point was d9pi. Average and standard error of three biological replicates are shown.

To investigate whether the classical *Bordetella* species can resist predation by amoebae, we performed a plaque assay in which *D. discoideum* was inoculated onto lawns of *B. bronchiseptica*, *B. parapertussis*_ov_, *B. parapertussis*_hu_, *B. pertussis or K. pneumoniae*. Plaques were formed in areas where *D. discoideum* consumed the bacteria and the area of plaque was measured and compared at different time points. Plaques formed on the lawns of *B. pertussis*, *B. parapertussis*_hu_ and *K. pneumoniae* at early time points, and the plaque size increased over time, as vegetative amoebae replicated and moved toward the surrounding bacteria as food source. In contrast, the plaques formed on the bacterial lawn of *B. bronchiseptica* and *B. parapertussis*_ov_ appeared only at late time points and were much smaller than those formed on lawns of *B. pertussis* and *B. parapertussis*_hu_ (**Figure 2**). The results indicate that the animal associated species *B. bronchiseptica* and *B. parapertussis*_ov_ can inhibit predation by the amoebae whereas the human-restricted *Bordetella* species have lost this ability.

### Human-Restricted *Bordetella* Species Fail to Survive in Fruiting Body

**Figure 3:**
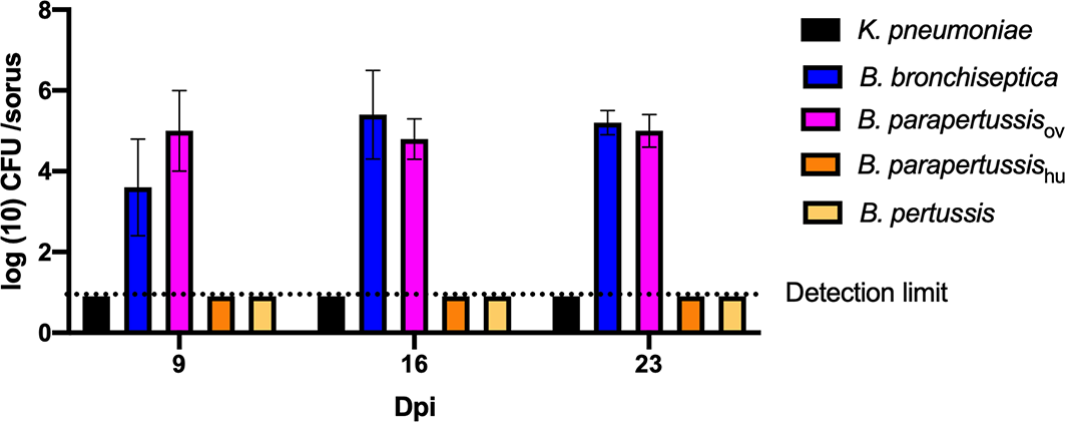
Human-restricted *Bordetella* species, *B. pertussis* and *B. parapertussis* human strain, fail to colonize sori. The sori formed on the lawn of *Bordetella* species were collected at days 9, 16 and 23 post inoculation of amoebae. The bacteria that survived inside sori were enumerated by plating samples on BG agar plates. The colony number will be counted post 48 hours incubation in 37°C. Error bars show the standard error of mean (n=3).

In our previous study (Taylor-Mulneix et al., 2017a), *B. bronchiseptica* was associated with the amoeba throughout the amoebic life cycle, translocated to fruiting body and traveled along with spores through multiple passages on other food sources. To test intra-sori survival of other classical *Bordetella* species, sori formed on lawns of different *Bordetella* species were harvested at various time points, and bacteria that survived inside the sori were enumerated as above. High numbers of *B. bronchiseptica* and *B. parapertussis*_ov_ were consistently recovered from sori across different time points, while neither *B. pertussis* nor *B. parapertussis*_hu_ were recovered from sori at any time point (**Figure 3**). The absence of *B. pertussis* and *B. parapertussis*_hu_ from sori indicates that human-restricted *Bordetella* species have lost (some of the) mechanisms involved in translocation to or survival within sori.

### Different Intracellular Survival Rates Observed Among Non-Classical *Bordetella* Species

**Figure 4:**
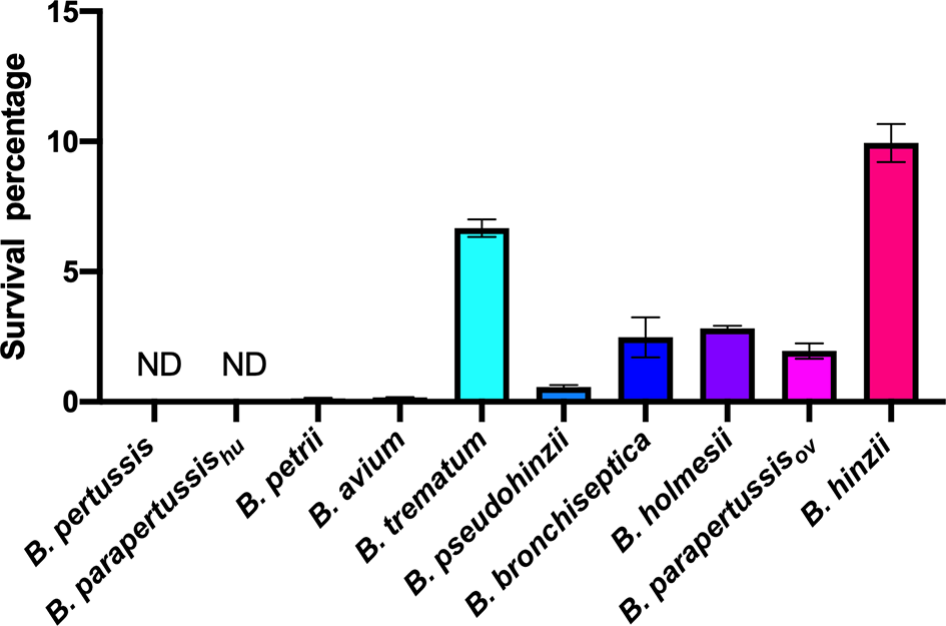
Different intracellular survival ratios observed within non-classical *Bordetella* species. Intracellular survival of *Bordetella* species inside vegetative amoebae was assessed in a 2-hour gentamicin protection assay using an MOI at 1. In this assay, various non-classical *Bordetella* species were tested, including *B. petrii*, *B. avium*, *B. trematum*, *B. pseudohinzii*, *B. holmesii* and *B. hinzii*. Error bars show the standard error of mean (n=3). ND, none detected.

We expanded the intracellular amoebic survival test to include 6 non-classical *Bordetella* species. In gentamicin protection assays, 7-10 percent of *B. trematum* and *B. hinzii* survived inside vegetative amoebae after 2 hours of treatment, while only around 0.1 percent of *B. avium* and *B. petrii* were recovered. Between these two groups, *B. parapertussis*_ov_, *B. bronchiseptica*, *B. pseudohinzii* and *B. holmesii* exhibited an intermediate recovery rate of 0.5 to 3% of the initially inoculated bacteria (**Figure 4**). In control groups without amoebae, more than 99.99% of each species were killed by the gentamicin (data not shown). These results showed that species isolated from non-human mammals have a relatively high intracellular survival rate in amoebae, suggesting that the ability to survive inside phagocytic cells remains important for these species.

### Some Non-Classical *Bordetella* Species Resist Predation by Amoebae

**Figure 5:**
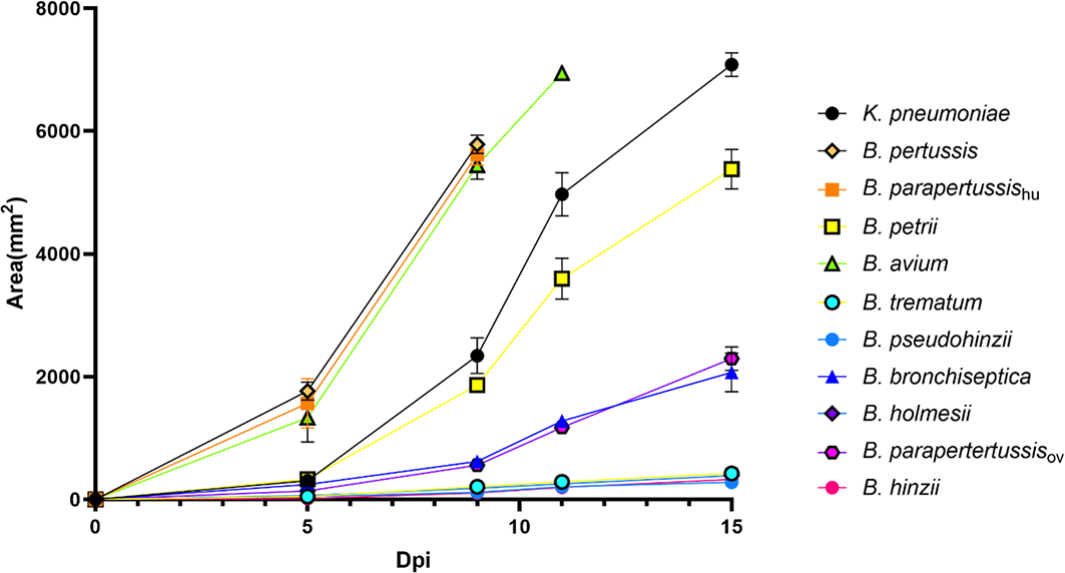
Non-classical *Bordetella* species showed different abilities to resist predation by amoebae. The plaques formed on the lawn of *Bordetella* species were measured at various time points post inoculation of amoebae. Because the plaques formed on the lawn of *B. pertussis*, *B. parapertussis_hu_* and *B. avium* covered the entire plate before d15pi, the last data were recorded at days d9pi and d11pi, respectively. Non-classical *Bordetella* species, including *B. petrii*, *B. avium*, *B. trematum*, *B. pseudohinzii*, *B. holmesii* and *B. hinzii*, were tested in this assay. The data are based on 3 replicates in each group, error bars show the standard error of mean.

To investigate the abilities of non-classical *Bordetella* species to resist amoebal predation, we carried out plaque formation assays by using bacterial lawns of classical and non-classical *Bordetella* species. The plaques formed on the bacterial lawns of *B. pertussis*, *B. parapertussis*_hu_, *B. avium* and *B. petrii* were similar in size to those on the lawn of *K. pneumoniae*, indicating that these species failed to resist predation by *D. discoideum*. In contrast, *B. hinzii*, *B. pseudohinzii*, *B. trematum* and *B. holmesii* successfully resisted predation by *D. discoideum*, as shown by the formation of very small plaques at late time points on the bacterial lawn of these species. Compared to these two groups, *B. bronchiseptica and B. parapertussis*_ov_ showed an intermediate ability to resist the predation from *D. discoideum*. The plaques formed on lawns of *B. bronchiseptica* and *B. parapertussis*_ov_ were larger than those of *B. hinzii*, *B. pseudohinzii*, *B. trematum* and *B. holmesii*, but far smaller than plaques on lawns of *B. pertussis*, *B. parapertussis*_hu_, *B. avium* or *B. petrii* (**Figure 5**). In addition, considering the bacterial growth rate may have an impact on resisting amoebal predation and plaque formation, we measured proliferation rates for all tested species (**Figure S1**). Species with comparable proliferation rates, like *B. pertussis, B. parapertussis*_hu_ and *B. holmesii*, showed distinctly different resistances to amoeba predation, suggesting bacterial growth rates have little, if any, effect on plaque formation. These results indicate that the ability to survive within cells is correlated to the resistance to amoebic predation. However, *B. bronchiseptica* and *B. parapertussis*_ov_ survived inside *D. discoideum* in relatively high numbers but did not inhibit plaque growth as efficiently as some of the non-classical *Bordetella* spp., suggesting there may be different mechanisms, potentially encoded by different sets of genes, involved in intracellular survival and inhibition of plaque formation.

### Some Non-Classical *Bordetella* Species Translocate to and Grow Within Fruiting Body

**Figure 6:**
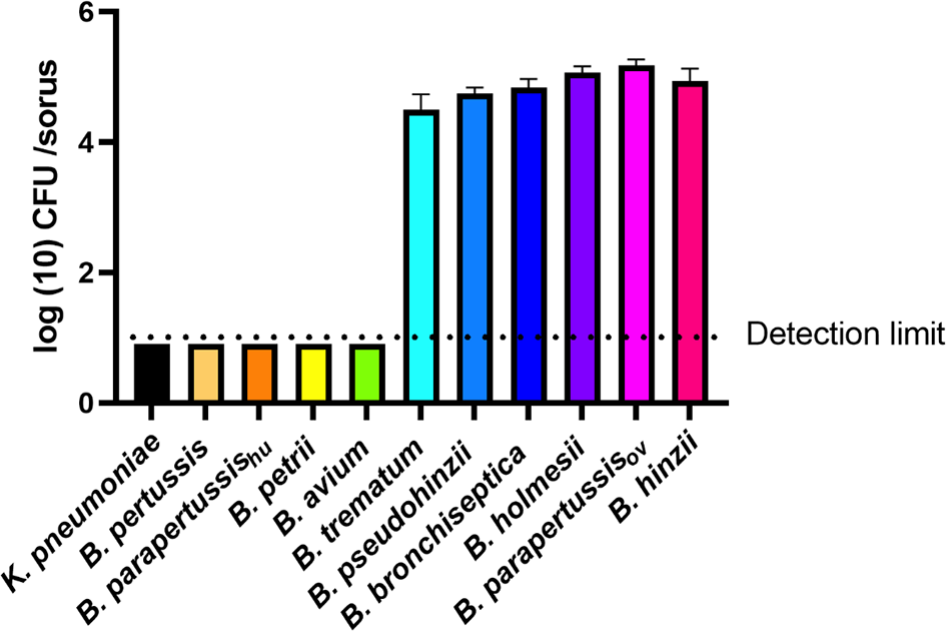
Some *Bordetella* species can survive inside sori. The sori formed on the lawn of *Bordetella* species were collected at day 16 post inoculation of amoebae. The bacteria that survived inside sori were enumerated by plating samples on BG agar plates. The colony number will be counted post 48 hours incubation in 37°C. The classical *Bordetella* species *B*. *bronchiseptica*, *B. pertussis* and *B. parapertussis* strains, and the non-classical species *B*. *petrii*, *B. avium*, *B*. *trematum*, *B*. *pseudohinzii*, *B. holmesii* and *B*. *hinzii*, were tested in this assay. The data are based on 3 replicates in each group, error bars show the standard error of mean.

*B. bronchiseptica* and *B. parapertussis*_ov_ efficiently translocated to fruiting body, while *B. pertussis* and *B. parapertussis*_hu_ failed to do so. To test if other *Bordetella* species can translocate to and survive or grow within fruiting body, and thereby be dispersed along with fruiting body, sori formed on bacterial lawns were collected at various time points and assessed for *Bordetella* spp. Among the tested non-classical *Bordetella* species, *B. hinzii*, *B. pseudohinzii*, *B. trematum*, and *B. holmesii* were recovered from sori at numbers similar to *B. bronchiseptica* and *B. parapertussis*_ov_, consistently yielding 10^4^ to 10^5^ bacteria per sorus. In contrast, *B. avium* and *B. petrii* failed to be recovered from collected sori (**Figure 6**). Thus, those species that failed to survive inside vegetative amoebae and to resist amoebic predation by inhibiting plaque expansion also lost the ability to translocate to fruiting body, suggesting that the three aspects of amoeba interaction we tested may positively correlate to each other.

### The Possible Genes That Contribute to Interactions With Amoeba

**Figure 7:**
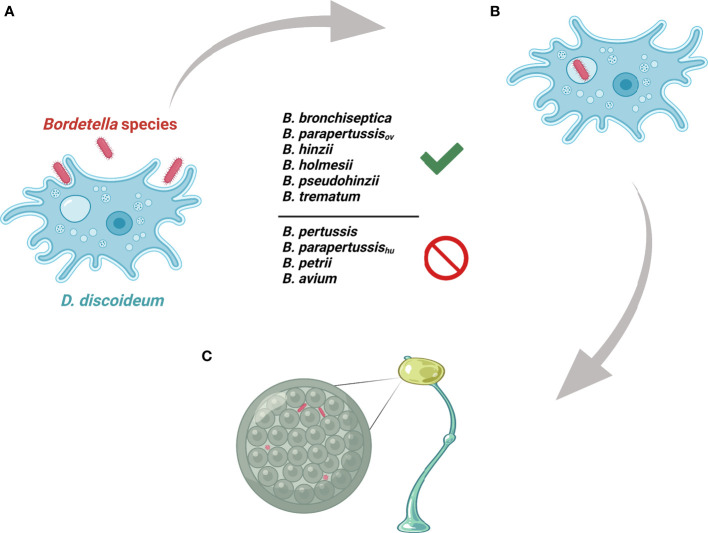
Illustration of *Bordetella*-amoebal interactions. **(A)** *D. discoideum* predates on *Bordetella* species. **(B)** Some *Bordetella* species resist phagocytosis of *D. discoideum*. **(C)** Some *Bordetella* species translocate to fruiting body.

**Figure 8:**
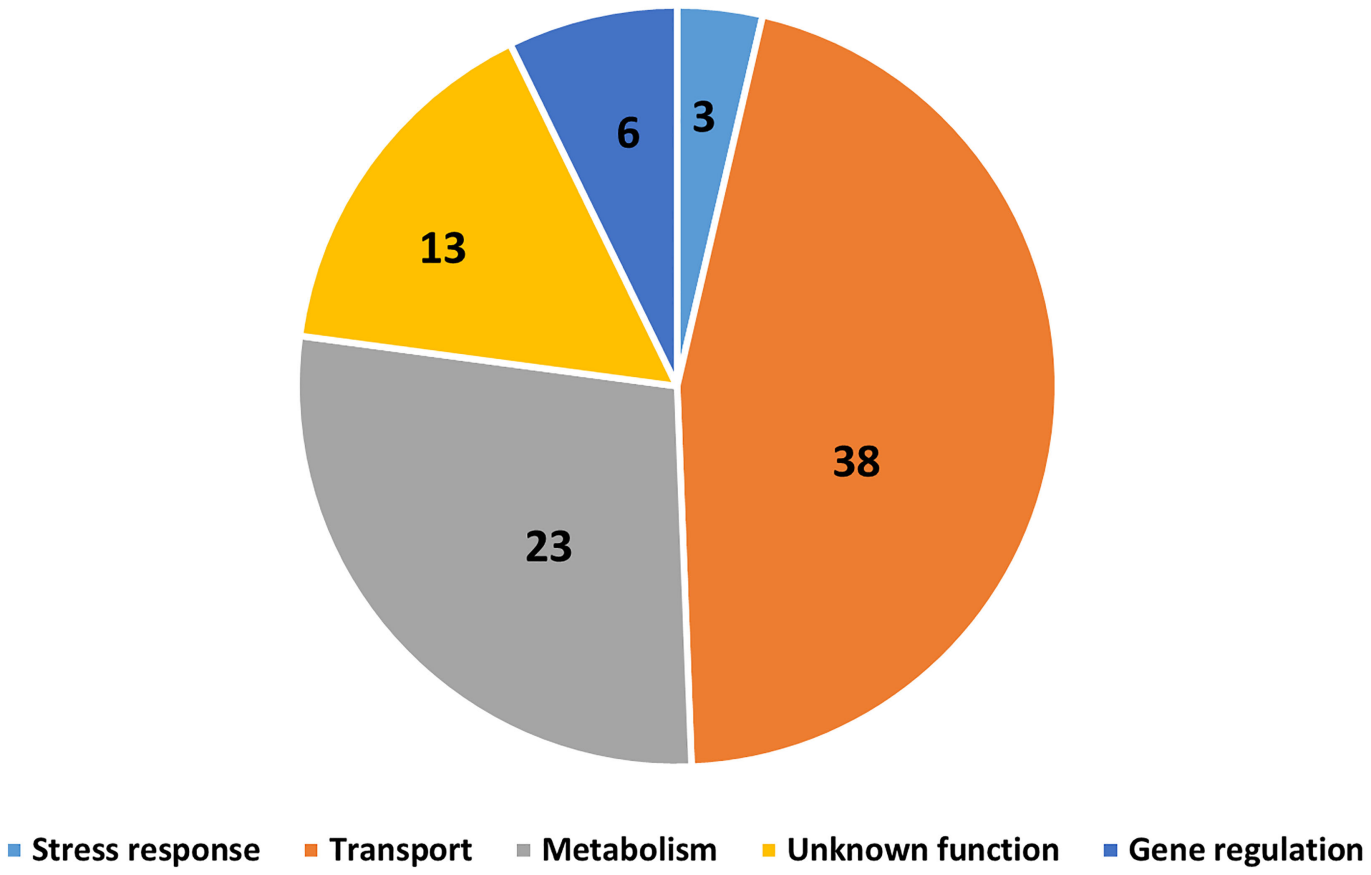
Gene candidates that potentially contribute to *Bordetella*-amoebal interactions. By using GWAS approach, 83 genes which may contribute to the interaction between *Bordetella* spp. and amoebae were identified. The genes are categorized based on their annotated functions being related to transport, metabolism, gene regulation, stress response, and unknown.

The *Bordetella* species *B. bronchiseptica*, *B. parapertussis*_ov_, *B. hinzii*, *B. pseudohinzii*, *B. holmesii* and *B. trematum* were able to survive inside vegetative cells, inhibit plaque expansion and translocate to sori, whereas *B. pertussis*, *B. parapertussis*_hu_, *B. petrii* and *B. avium* were not (**Figure 7**). To probe the genes that might be involved in interactions of *Bordetella* with amoebae, GWAS was used to identify potential target genes. Genes that were present in *B. bronchiseptica*, *B. parapertussis*_ov_, *B. holmesii*, *B. hinzii*, *B. pseudohinzii* and *B. trematum* but were absent or degraded in any species that failed to interact with amoebae were considered. Furthermore, to probe the genes that possibly contribute to survival inside vegetative *D. discoideum*, we evaluated the transcriptional profile of *B. bronchiseptica* inside *D. discoideum* cells in comparison to *B. bronchiseptica* cultured in medium. The genes that showed different expression under these two conditions are included in this selection. Based on these criteria, we identified 83 genes that are annotated as involved in metabolism, regulation of gene expression (transcriptional regulators), stress response, or unknown functions (**Figure 8** and **Table S1**).

### The Correlation Between *Bordetella*-Amoebal Interaction and Mammalian Cell Intracellular Survival

**Figure 9:**
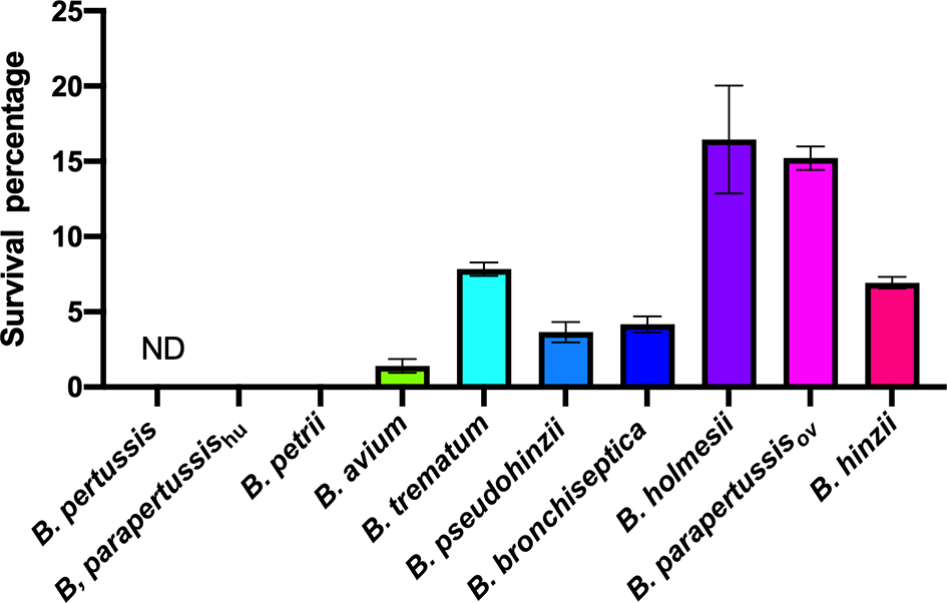
Different intracellular survival rates of non-classical *Bordetella* species in RAW 264.7 murine macrophages. Intracellular survival of *Bordetella* species inside murine RAW macrophages was assessed in a 2-hour gentamicin protection assay at an MOI of 1. There were 3 replicates in each group. Error bars show the standard error of mean. ND, none detected.

**Figure 10:**
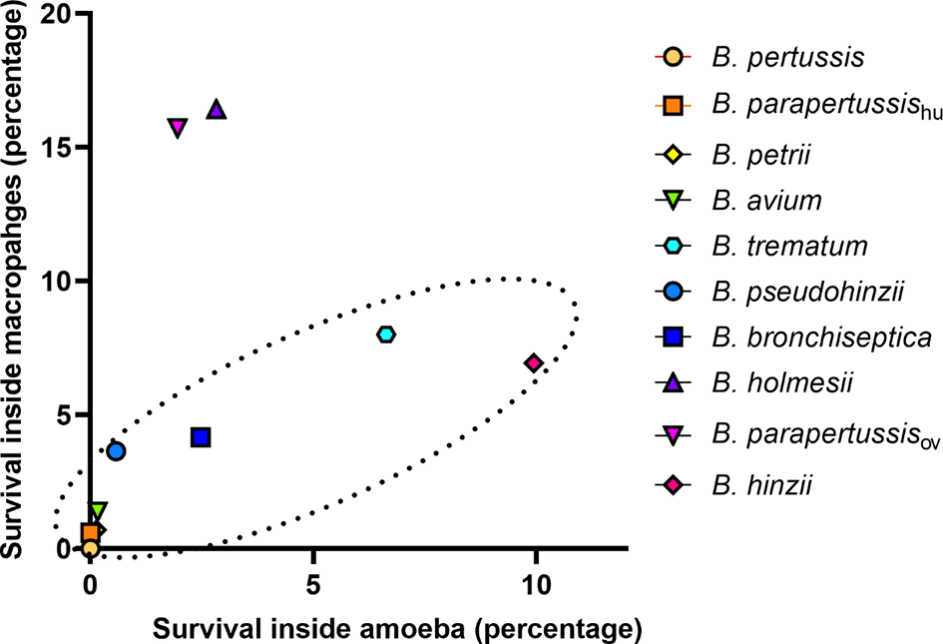
Correlation between *Bordetella*-amoebal interaction and mammalian cell intracellular survival. The survival percentage inside sori is located on the X axis and the survival percentage inside murine RAW 264.7 macrophages located on the Y axis. The *Bordetella* species highlighted within the dash oval have a correlation coefficient value at 0.889035 for these two variables.

Bacteria-amoeba interactions are hypothesized to represent an important step in the evolution of *Bordetella* spp. from environmental microbes to mammalian pathogens, based on the prediction that mechanisms effective against amoebal predation also protect against phagocytes of the mammalian immune system. To test the correlation between *Bordetella*-amoeba interactions and mammalian cell intracellular survival of *Bordetella* species, we mixed each with murine macrophages for 1 hour before adding gentamicin to kill extracellular bacteria, then quantified the surviving intracellular bacteria. Around 15% of *B. holmesii* and *B. parapertussis*_ov_, and 5-10% of *B. bronchiseptica*, *B. pseudohinzii*, *B. hinzii* and *B. trematum* were recovered from inside macrophages. In contrast, much smaller numbers of *B. parapertussis*_hu_, *B. petrii* and *B. avium*, and zero *B. pertussis* were recovered (**Figure 9**). To assess whether intracellular survival inside amoebae correlates with survival in murine macrophages, we plotted the survival of *Bordetella* in unicellular amoebae against survival in RAW macrophages. The correlation coefficient (*R* ^2^ = 0.889035) suggests that *Bordetella* species that survive inside amoebae are highly likely to also survive and persist inside macrophages. Two outliers, *B. parapertussis*_ov_ and *B. holmesii*, displayed an intermediate ability to survive inside amoebae but survive well inside murine macrophages with a much higher efficiency compared to other species (**Figure 10**), suggesting some specialization to interactions with phagocytes of their mammalian host.

## Discussion

*Bordetella* species have been shown to survive inside various mammalian cell types (Steed et al., 1991; Friedman et al., 1992; Guzman et al., 1994; Lamberti et al., 2010; Lamberti et al., 2013). The capacity of *B. bronchiseptica* to survive inside soil amoebae implies that the interaction of *Bordetella* species and soil amoebae may have served as an important evolutionary step for the bacterial adaptation to interaction with and survival inside mammalian cells (Taylor-Mulneix et al., 2017a; Taylor-Mulneix et al., 2017b). However, throughout the evolution of genus *Bordetella*, many genes have been lost and gained (Linz et al., 2016), and whether the mechanisms that allow *Bordetella* spp. to interact with soil amoebae are conserved across the genus is unknown. Herein, we tested 9 *Bordetella* species in their interactions with *D. discoideum*. The animal-associated *B. bronchiseptica*, *B. hinzii*, *B. pseudohinzii*, *B. trematum*, and *B. parapertussis*_ov_, as well as the human-associated *B. holmesii*, survived inside vegetative amoebae, resisted predation by inhibition of plaque expansion, and translocated to the sori to disseminate along with fruiting body (**Figures 4**–**6**). This ability of these species to interact with amoebae likely facilitates a lifestyle that interconnects colonization and spread among the animal host(s) with propagation in the soil amoeba as an environmental niche, as we previously described for *B. bronchiseptica* (Taylor-Mulneix et al., 2017a).

In contrast to the above species, *B. pertussis*, *B. parapertussis*_hu_, and *B. avium*, all of which have evolved and adapted to a closed life cycle circulating within a specific host population (Dewan and Harvill, 2019), have lost the ability to interact with *D. discoideum* and to use it as an environmental reservoir. While the classical *Bordetella* species are closely related and share many virulence factors (Linz et al., 2016), only *B. bronchiseptica* and *B. parapertussis*_ov_ were able to interact with *D. discoideum* in all three assays. *B. parapertussis_hu_* and *B. pertussis* independently originated and evolved from a *B. bronchiseptica*-like ancestor (Parkhill et al., 2003; Diavatopoulos et al., 2005). Their evolution and host specialization was accompanied by large scale gene loss that was facilitated by homologous recombination between multiple copies of IS elements IS*481* in *B. pertussis* and IS*1002* in *B. parapertussis* (Parkhill et al., 2003), which apparently resulted in the loss of genes necessary for the interaction with amoeba and thus in loss of the extra-host environmental niche for these human-restricted pathogens. Interestingly, *B. parapertussis*_ov_, which is only known to infect sheep, retained the ability to interact with *D. discoideum*, suggesting that it either may not have fully committed to a closed life cycle in sheep, or that its ability to thrive in an environmental reservoir is crucial for survival of the species. (Re-)Infection of sheep grazing on previously grazed pastures may be important for optimal success in this relatively short-lived host or for transmission between populations (flocks). Within the target genes identified for *Bordetella*-amoebae interactions, a few of them are differentially expressed between two *B. parapertussis* strains, including hydrolase, thiamine biosynthesis lipoprotein ApnE and phosphonate ABC transporter which all relate to bacterial metabolism, suggesting that a switch of metabolic state may be required for resisting amoebic predation.

Just like *B. pertussis*, *B. holmesii* is only known to colonize and infect humans, and this specialization to a closed human-to-human lifecycle was also accompanied by genome reduction. Based on this observation, we previously predicted that, similar to *B. pertussis*, the human-associated *B. holmesii* will likely fail to evade amoebic predation and to hijack the amoebic life cycle (Taylor-Mulneix et al., 2017b). However, despite a drastic genome reduction during its evolution, which is emphasized by a genome size of approximately 3.7 Mb compared to the 4.9 Mb of its closest relative *B. hinzii* (Linz et al., 2016), *B. holmesii* successfully interacted with *D. discoideum* in all three assays; *B. holmesii* persisted inside vegetative amoebae, inhibited expansion of the amoeba plaque, and was recovered from fruiting body in high numbers (**Figures 4**–**6**). Thus, the gene loss associated with the origin and evolution of this emerging pathogen did apparently not affect genes whose products are necessary to hijack the amoeba life cycle, and the *B. holmesii* transmission cycle in humans may not be as closed as we previously thought. Instead, the *B. holmesii*–amoeba interaction that we observed under laboratory conditions reveals the possibility of a true *B. holmesii*-amoeba relationship in nature. Thus, there might be an environmental niche for this emerging human pathogen in amoeba, which constitutes a potential source for infection.

We previously presented a hypothetical model that illustrates the evolution from environmental microbes to obligate human-restricted pathogens. In this model, (A) most environmental bacteria represent a food source for amoebae. (B) Stimulated by selective pressure of environmental predators, a subset of those bacteria developed resistance to digestion by amoebic phagocytes. (C) Opportune exposure to higher animals favored those already evolved to evade phagocytic killing, allowing further evolution to persist in animal hosts while retaining the ability to interact with and utilize the amoebic host as an environmental niche. (D) Finally, specialization to efficiently transmit amongst animal hosts in a successful closed life cycle allowed for genome reduction including loss of the dispensable genes mediating amoeba interactions (Taylor-Mulneix et al., 2017b). Humans represent a uniquely large, long-lived and well-connected population, readily allowing success of such specialized pathogens. *Bordetella* species associated with other animals tend to retain the ability to hijack and manipulate the amoeba growth and maturation, consistent with the view that environmental survival might still contribute to their success, potentially mediating persistence over time or transmission to new populations. Somewhat surprisingly, *B. petrii*, the only species tested that was originally isolated from an environmental source (von Wintzingerode et al., 2001), failed to interact with *D. discoideum* in all three assays, showing the same phenotype as the control species *K. pneumoniae* which represented a mere amoeba food source. This suggests a different lifestyle for *B. petrii* compared to most analyzed animal-pathogenic species and opportunistic human pathogens. Based on our previously published data that *B. petrii* is located closer to the root of the phylogenetic tree and thus from an evolutionary perspective older than the animal-associated *Bordetella* species (Linz et al., 2016; Hamidou Soumana et al., 2017), this may indicate that the *B. petrii* lineage did not acquired the means to resist amoebic predation, or that it subsequently specialized to an unknown niche that does not require those genes.

Which genes contribute to interactions with amoebae? We used an GWAS approach to identify potential target genes by selecting those that are present and apparently functional in *Bordetella* species that interact successfully with amoebae but are absent or predicted to be inactive in the species that failed to. 83 genes were identified by this approach, including 38 transporter genes, 23 metabolic genes, 6 regulatory genes, 3 stress response genes and 13 genes with unknown functions. Bacteria have been reported to evade amoebic predation by inhibiting phagolysosome infusion or increasing their resistance to acidic environments inside phagolysosome (Bozue and Johnson, 1996; La Scola and Raoult, 2001; Greub and Raoult, 2004). Here, *Bordetella* spp. may increase secretion of virulence factors which block phagolysosome fusion, during which genes related to transporter and gene regulation would be upregulated. Additionally, the expression of stress response genes may enhance the capacity of bacteria to survive inside this extreme environment, as has been observed for *B. bronchiseptica* inside macrophages (Rivera et al., 2019). Furthermore, *Bordetella* spp. are well documented to use different metabolic strategies to cope with extreme changes in their surroundings, particularly in response to changes in temperature, pH, or nutrient availability (Rivera et al., 2019). *Bordetella* species that displayed a high survival level inside vegetative amoebae also survived better inside mammalian phagocytes, suggesting a potential overlap of genes mediating interactions with amoebae and host phagocytic immune cells. Better understanding how *Bordetella* species resist amoebal predation may reveal promising targets for interventions to treat and prevent the important diseases they cause.

## Data Availability Statement

The datasets presented in this study can be found in online repositories. The names of the repository/repositories and accession number(s) can be found below: https://www.ncbi.nlm.nih.gov/geo/, GSE190363.

## Author Contributions

LM, BL and EH conceived the study. LM, BL, AC, KD, IR and EH designed the experiments. LM, BL, AC and IR performed the experiments. LM, BL, AC, KD, IR and EH analyzed the data. LM, BL, KD and EH wrote the manuscript. All authors contributed to the article and approved the submitted version.

## Funding

This work was supported by grants AI156293, DC018496, AI159347, AI149787, AI142678, GM113681 from the National Institutes of Health to EH. The funders had no role in the study design, data collection and interpretation, or the decision to submit the work for publication.

## Conflict of Interest

The authors declare that the research was conducted in the absence of any commercial or financial relationships that could be construed as a potential conflict of interest.

## Publisher’s Note

All claims expressed in this article are solely those of the authors and do not necessarily represent those of their affiliated organizations, or those of the publisher, the editors and the reviewers. Any product that may be evaluated in this article, or claim that may be made by its manufacturer, is not guaranteed or endorsed by the publisher.

## Supplementary Material

Supplementary Figure 1 Growth curves of *Bordetella* species at 21°C.

Supplementary Table 1 Genes associated with *Bordetella*-amoebal interactions.
