# Supplementary figures and images for: Natural History and Ecology of Interactions Between Bordetella Species and Amoeba
Source: Front Cell Infect Microbiol. 2022 Feb 9;12:798317. doi: 10.3389/fcimb.2022.798317 (PMC8863592; doi:10.3389/fcimb.2022.798317)

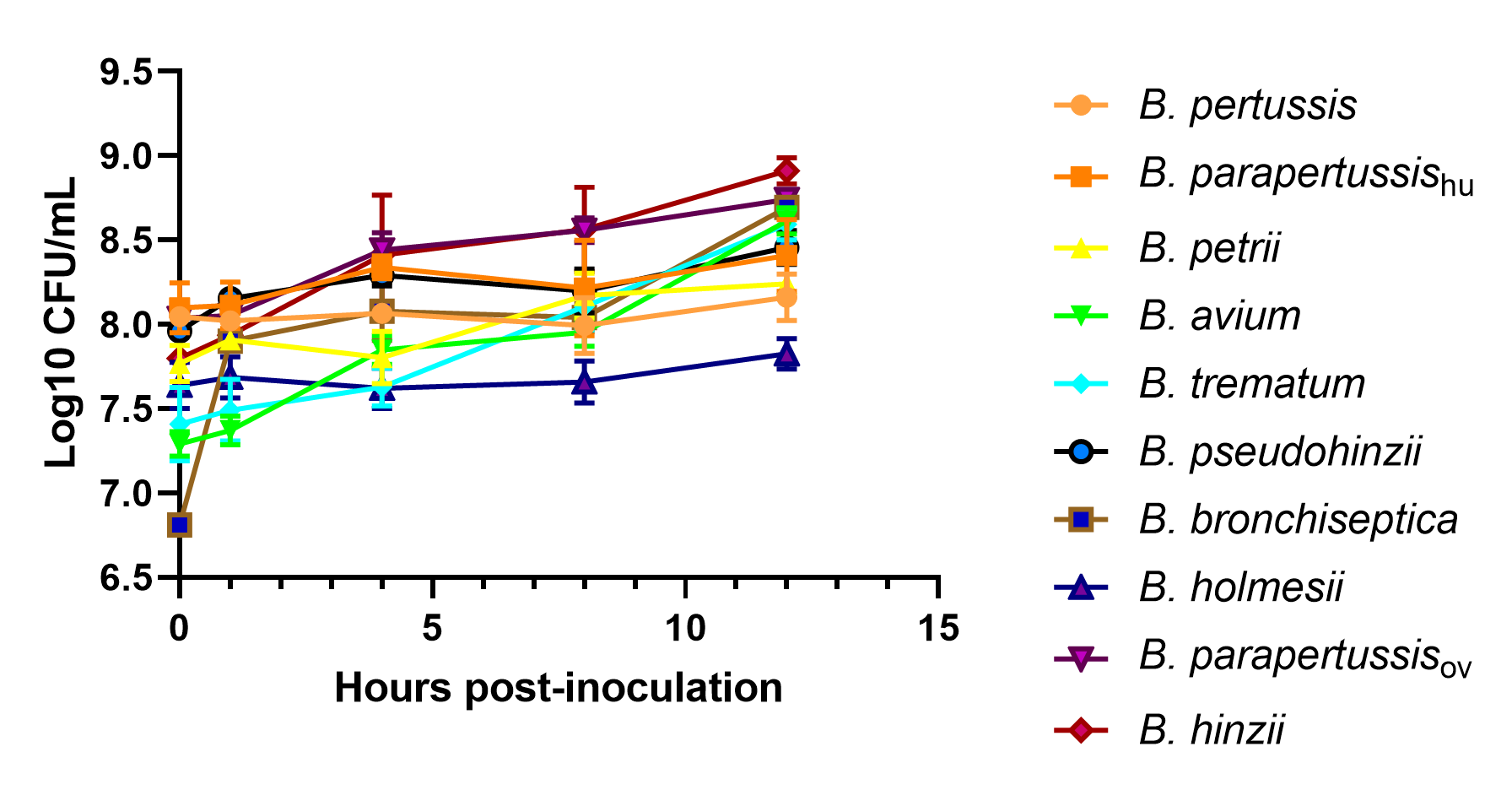

Supplement: Supplementary Figure 1 — Growth curves of Bordetella species at 21°C. [file Image_1.tif]
