# Supplementary material for: Natural History and Ecology of Interactions Between Bordetella Species and Amoeba
Source: Front Cell Infect Microbiol. 2022 Feb 9;12:798317. doi: 10.3389/fcimb.2022.798317 (PMC8863592; doi:10.3389/fcimb.2022.798317)
Supplement: Supplementary Table 1 — Genes associated with Bordetella-amoebal interactions. [file Table_1.docx]

| *B. bronchiseptica* |  | Gene presence | | | | *B. bronchiseptica* RB50 protein similarity with | | | | | |
| --- | --- | --- | --- | --- | --- | --- | --- | --- | --- | --- | --- |
| **Locus_tag** | **product** | ***B. bronchiseptica*** | ***B. parapertusssi* ovine** | ***B. parapertussis* human** | ***B. pertusssi*** | ***B. hinzii*** | ***B. pseudohinzii*** | ***B. avium*** | ***B. petrii*** | ***B. trematum*** | ***B. holmsii*** |
| BB0039 | hydrolase | **+** | **+** | **Stop** | **+** | 0.683 | 0.676 | 0.355 | 0.375 | 0.721 | 0.669 |
| BB0163 | ABC transporter permease MlaE | **+** | **+** | **+** | **+** | 0.759 | 0.767 | 0.767 | 0.222 | 0.77 | 0.749 |
| BB0164 | ABC transporter ATPase MlaF | **+** | **+** | **+** | **+** | 0.778 | 0.774 | 0.778 | 0.344 | 0.771 | 0.789 |
| BB0165 | ABC transporter periplasmic MlaD | **+** | **+** | **+** | **FS** | 0.629 | 0.622 | 0.641 | 0 | 0.644 | 0.619 |
| BB0215 | ferric uptake regulator | **+** | **+** | **+** | **-** | 0.63 | 0.603 | 0.452 | 0.445 | 0.623 | 0.569 |
| BB0216 | hypothetical protein | **+** | **+** | **+** | **-** | 0.515 | 0.508 | 0 | 0.439 | 0.508 | 0.508 |
| BB0224 | ArsR family regulator | **+** | **+** | **+** | **-** | 0.73 | 0.739 | 0.324 | 0.315 | 0.667 | 0.685 |
| BB0388 | Periplasmic aromatic solutes transporter | **+** | **+** | **+** | **+** | 0.533 | 0.539 | 0.621 | 0.4 | 0.521 | 0.536 |
| BB0886 | Periplasmic aromatic solutes transporter | **+** | **+** | **+** | **+** | 0.591 | 0.591 | 0.604 | 0 | 0.557 | 0.615 |
| BB0898 | hypothetical protein | **+** | **+** | **+** | **+** | 0.793 | 0.797 | 0.747 | 0 | 0.731 | 0.782 |
| BB0945 | Phosphonate ABC transport permease | **+** | **+** | **+** | **+** | 0.858 | 0.853 | 0 | 0.85 | 0.842 | 0.824 |
| BB0946 | Phosphonate ABC transport permease | **+** | **+** | **+** | **+** | 0.791 | 0.747 | 0 | 0.801 | 0.777 | 0.723 |
| BB0947 | Phosphonate ABC transport ATP-binding | **+** | **FS** | **+** | **Trunc** | 0.875 | 0.887 | 0.375 | 0.848 | 0.871 | 0.867 |
| BB0948 | Phosphonate ABC transport periplasmic | **+** | **+** | **+** | **+** | 0.863 | 0.866 | 0 | 0.863 | 0.896 | 0.907 |
| BB0950 | haloacid dehalogenase-like hydrolase | **+** | **+** | **+** | **+** | 0.792 | 0.781 | 0 | 0.849 | 0.774 | 0.811 |
| BB1143 | CoA-transferase | **+** | **+** | **+** | **-** | 0.869 | 0.543 | 0.415 | 0.421 | 0.741 | 0.867 |
| BB1352 | aldo/keto reductase | **+** | **+** | **+** | **+** | 0.684 | 0.678 | 0.657 | 0.313 | 0.636 | 0.651 |
| BB1384 | acetate kinase | **+** | **+** | **+** | **+** | 0.758 | 0.755 | 0.765 | 0 | 0.768 | 0.742 |
| BB1385 | phosphate acetyl/butaryl transferase | **+** | **+** | **+** | **+** | 0.83 | 0.84 | 0.811 | 0.176 | 0.811 | 0.805 |
| BB1442 | ABC transport periplasmic protein | **+** | **+** | **+** | **-** | 0.87 | 0.876 | 0.237 | 0.873 | 0.806 | 0.855 |
| BB1443 | ABC transporter ATPase | **+** | **+** | **+** | **-** | 0.896 | 0.892 | 0.401 | 0.903 | 0.874 | 0.874 |
| BB1444 | ABC transporter permease | **+** | **+** | **+** | **-** | 0.928 | 0.924 | 0.364 | 0.92 | 0.902 | 0.898 |
| BB1737 | acetyl-coenzyme A carboxylase carboxyl transferase subunit alpha | **+** | **+** | **+** | **+** | 0.928 | 0.928 | 0.931 | 0.112 | 0.925 | 0.922 |
| BB1897 | phosphohydrolase | **+** | **+** | **+** | **+** | 0.783 | 0.767 | 0.762 | 0 | 0.735 | 0.772 |
| BB1947 | N-formylglutamate amidohydrolase | **+** | **+** | **+** | **+** | 0.832 | 0.832 | 0.241 | 0.804 | 0.77 | 0.818 |
| BB1948 | glutamate transport periplasmic receptor | **+** | **+** | **+** | **+** | 0.836 | 0.833 | 0.457 | 0.827 | 0.823 | 0.845 |
| BB1949 | LysR-family transcriptional regulator | **+** | **+** | **+** | **+** | 0.879 | 0.879 | 0.422 | 0.869 | 0.827 | 0.869 |
| BB1999 | Periplasmic aromatic solutes transporter | **+** | **+** | **+** | **Trunc** | 0.818 | 0.787 | 0.441 | 0.468 | 0.732 | 0.772 |
| BB2000 | aldolase | **+** | **+** | **+** | **+** | 0.81 | 0.806 | 0.488 | 0.845 | 0.841 | 0.798 |
| BB2157 | osmotically inducible protein C | **+** | **+** | **+** | **FS** | 0.766 | 0.759 | 0.177 | 0.22 | 0.674 | 0.752 |
| BB2160 | ectoine/hydroxyectoine ABC transporter substrate-binding protein EhuB | **+** | **+** | **+** | **+** | 0.663 | 0.677 | 0.19 | 0.764 | 0.621 | 0.647 |
| BB2161 | ectoine/hydroxyectoine ABC transporter permease subunit EhuC | **+** | **+** | **+** | **+** | 0.765 | 0.751 | 0.381 | 0.657 | 0.714 | 0.751 |
| BB2162 | ectoine/hydroxyectoine ABC transporter permease subunit EhuD | **+** | **+** | **+** | **+** | 0.83 | 0.848 | 0.296 | 0.848 | 0.786 | 0.798 |
| BB2163 | ATP-binding component of ABC transporter | **+** | **+** | **+** | **+** | 0.863 | 0.867 | 0.496 | 0.871 | 0.836 | 0.816 |
| BB2252 | hypothetical protein | **+** | **+** | **+** | **+** | 0.833 | 0.838 | 0.812 | 0 | 0.812 | 0.833 |
| BB2391 | DUF445 protein | **+** | **+** | **+** | **+** | 0.701 | 0.724 | 0 | 0 | 0.712 | 0.701 |
| BB2501 | amino-acid ABC transporter, APT-binding | **+** | **+** | **+** | **+** | 0.793 | 0.785 | 0.469 | 0.742 | 0.695 | 0.738 |
| BB2502 | amino-acid ABC transporter, permease | **+** | **+** | **+** | **+** | 0.907 | 0.894 | 0.327 | 0.916 | 0.832 | 0.872 |
| BB2503 | amino-acid ABC transporter, periplasmic | **+** | **+** | **+** | **+** | 0.752 | 0.756 | 0.317 | 0.786 | 0.762 | 0.798 |
| BB2885 | DUF1800 protein | **+** | **+** | **+** | **FS** | 0.532 | 0.56 | 0 | 0 | 0.513 | 0.517 |
| BB2886 | DUF1501 protein | **+** | **+** | **+** | **+** | 0.684 | 0.674 | 0 | 0 | 0.643 | 0.674 |
| BB3121 | DUF4377 protein | **+** | **+** | **+** | **-** | 0.617 | 0.609 | 0 | 0.399 | 0.56 | 0.584 |
| BB3211 | aspartate 1-decarboxylase precursor | **+** | **+** | **+** | **+** | 0.943 | 0.943 | 0.975 | 0 | 0.943 | 0.943 |
| BB3226 | Uncharacterized membrane protein YeiB | **+** | **+** | **+** | **+** | 0.558 | 0.568 | 0 | 0 | 0.533 | 0.516 |
| BB3279 | PEPSY-like protein | **+** | **+** | **+** | **+** | 0.776 | 0.781 | 0 | 0.694 | 0.78 | 0.757 |
| BB3280 | DUF2270 protein | **+** | **+** | **+** | **+** | 0.801 | 0.813 | 0 | 0.854 | 0.731 | 0.813 |
| BB3281 | DUF4198 protein | **+** | **+** | **+** | **+** | 0.718 | 0.722 | 0 | 0.748 | 0.673 | 0.699 |
| BB3282 | thiamine biosynthesis lipoprotein ApbE | **+** | **+** | **FS** | **+** | 0.589 | 0.609 | 0 | 0.586 | 0.565 | 0.586 |
| BB3283 | oxidoreductase | **+** | **+** | **+** | **+** | 0.54 | 0.538 | 0.083 | 0.632 | 0.538 | 0.558 |
| BB3358 | Periplasmic aromatic solutes transporter | **+** | **+** | **+** | **+** | 0.799 | 0.796 | 0.437 | 0.433 | 0.693 | 0.78 |
| BB3359 | malonyl-CoA synthetase | **+** | **+** | **+** | **+** | 0.844 | 0.836 | 0.33 | 0.85 | 0.811 | 0.803 |
| BB3360 | methylmalonyl-CoA decarboxylase | **+** | **+** | **+** | **+** | 0.649 | 0.631 | 0.295 | 0.683 | 0.679 | 0.627 |
| BB3361 | malonyl-CoA decarboxylase | **+** | **+** | **+** | **+** | 0.749 | 0.741 | 0 | 0.862 | 0.732 | 0.73 |
| BB3362 | gntR-family transcriptional regulator | **+** | **+** | **+** | **+** | 0.701 | 0.709 | 0.276 | 0.72 | 0.622 | 0.673 |
| BB3409 | acyl-CoA dehydrogenase | **+** | **+** | **+** | **+** | 0.712 | 0.712 | 0.163 | 0.744 | 0.662 | 0.662 |
| BB3850 | betaine/carnitine/choline transporter | **+** | **+** | **+** | **+** | 0.717 | 0.716 | 0.709 | 0 | 0.708 | 0.697 |
| BB4017 | osmotically inducible lipoprotein B | **+** | **+** | **+** | **+** | 0.877 | 0.877 | 0.822 | 0.342 | 0.808 | 0.863 |
| BB4028 | periplasmic transport protein | **+** | **+** | **+** | **+** | 0.889 | 0.891 | 0.889 | 0.432 | 0.882 | 0.891 |
| BB4187 | Periplasmic aromatic solutes transporter | **+** | **+** | **+** | **+** | 0.758 | 0.768 | 0.379 | 0.404 | 0.722 | 0.743 |
| BB4236 | TolA translocation protein | **+** | **+** | **+** | **+** | 0.691 | 0.668 | 0.306 | 0.288 | 0.675 | 0.539 |
| BB4284 | DUF533 protein | **+** | **+** | **+** | **+** | 0.788 | 0.765 | 0.695 | 0 | 0.73 | 0.637 |
| BB4285 | Periplasmic aromatic solutes transporter | **+** | **+** | **+** | **+** | 0.699 | 0.684 | 0.636 | 0.425 | 0.693 | 0.678 |
| BB4523 | Anion permease ArsB/NhaD | **+** | **+** | **+** | **+** | 0.849 | 0.847 | 0.821 | 0 | 0.851 | 0.855 |
| BB4524 | L-lactate dehydrogenase | **+** | **+** | **+** | **+** | 0.786 | 0.772 | 0.772 | 0.336 | 0.766 | 0.766 |
| BB4537 | universal stress protein | **+** | **+** | **+** | **+** | 0.771 | 0.774 | 0.733 | 0.149 | 0.767 | 0.729 |
| BB4556 | membrane efflux protein | **+** | **+** | **+** | **+** | 0.781 | 0.778 | 0 | 0 | 0.731 | 0.751 |
| BB4559 | Serine aminopeptidase | **+** | **+** | **+** | **Trunc** | 0.588 | 0.567 | 0 | 0.141 | 0.534 | 0.563 |
| BB4593 | Succinylglutamate desuccinylase | **+** | **+** | **+** | **+** | 0.718 | 0.683 | 0.308 | 0.7 | 0.683 | 0.671 |
| BB4637 | fatty acid hydroxylase | **+** | **+** | **+** | **-** | 0.761 | 0.764 | 0.737 | 0 | 0.767 | 0.752 |
| BB4638 | polysaccharide deacetylase | **+** | **+** | **+** | **-** | 0.752 | 0.744 | 0.733 | 0 | 0.74 | 0.713 |
| BB4639 | alcohol dehydrogenase YncE | **+** | **+** | **+** | **+** | 0.775 | 0.769 | 0.792 | 0 | 0.745 | 0.757 |
| BB4660 | DUF523 protein | **+** | **+** | **+** | **+** | 0.669 | 0.651 | 0.62 | 0 | 0.663 | 0.602 |
| BB4950 | DUF1653 protein | **+** | **+** | **+** | **+** | 0.753 | 0.753 | 0.753 | 0 | 0.765 | 0.728 |
| BB4954 | periplasmic binding protein | **+** | **+** | **+** | **+** | 0.897 | 0.884 | 0.091 | 0.909 | 0.85 | 0.853 |
| BB4955 | periplasmic transporter DctM | **+** | **+** | **+** | **+** | 0.902 | 0.899 | 0.159 | 0.895 | 0.862 | 0.884 |
| BB4961 | ABC transporter, periplasmic binding protein | **+** | **+** | **+** | **+** | 0.794 | 0.791 | 0.771 | 0.201 | 0.768 | 0.772 |
| BB4962 | ABC transporter, permease | **+** | **+** | **+** | **+** | 0.833 | 0.841 | 0.825 | 0.416 | 0.805 | 0.802 |
| BB5008 | TCS sensor kinase | **+** | **+** | **+** | **+** | 0.769 | 0.766 | 0.354 | 0.798 | 0.752 | 0.752 |
| BB5009 | Tripartite-type tricarboxylate receptor TctC | **+** | **+** | **+** | **+** | 0.88 | 0.883 | 0.281 | 0.886 | 0.856 | 0.859 |
| BB5010 | Tripartite tricarboxylate transporter TctB | **+** | **+** | **+** | **+** | 0.828 | 0.814 | 0 | 0.828 | 0.793 | 0.779 |
| BB5011 | Tripartite tricarboxylate transport receptor TctA | **+** | **+** | **+** | **+** | 0.905 | 0.909 | 0.381 | 0.905 | 0.907 | 0.909 |
